# Letters to the Editor

**DOI:** 10.1097/01.NPR.0000000000000407

**Published:** 2026-01-22

**Authors:** Julee Briscoe Waldrop, Staci S. Reynolds

**Affiliations:** **Julee Briscoe Waldrop** is Professor Emeritus, The University of North Carolina at Chapel Hill School of Nursing and Consulting Associate, Duke University School of Nursing.; **Staci S. Reynolds** is Clinical Professor, Duke University School of Nursing.

**Dear Editor,**

Regarding the article “Promoting top-of-degree APRN practice through PhD/DNP partnerships,”^1^ we appreciate the author's clarity on distinctions between the DNP and the PhD degrees and their recognition that the potential of the DNP degree in practice is still largely unrealized, especially for advanced practice registered nurses (APRNs). However, we disagree with the statement that “to truly maximize collaboration, DNP/PhD synergistic partnerships may be realized in the pursuit of implementation science, with each role practicing to the full scope of their training.” Implementation science is the scientific study of methods and strategies that facilitate the uptake of evidence-based practices (EBP) and research into regular use by clinicians.^2^,^3^

Implementation science is a type of *research* to determine, for example, which implementation strategies are most effective (such as conducting a randomized controlled trial to see if educational outreach visits versus audit and feedback are more effective at translating evidence into practice). The DNP curriculum/degree does not provide graduates with the skills to conduct this type of empirical research. Rather, the DNP degree should be preparing graduates to be leaders in EBP and quality improvement (QI) where they search, appraise, and synthesize the evidence on *best practices* as well as *effective implementation strategies*, and then use iterative tests of change tailored to the local context (QI) to translate these EBPs into practice. Graduates should be using findings from implementation science research to strengthen the rigor of their EBPQI initiatives by ensuring *evidence-based practices* are implemented using *evidence-based strategies*. Using findings from implementation science research is not the same as *conducting* implementation science research, and reports suggesting this adds further confusion to the differences between the PhD and DNP degree preparations. When health care leaders are continuously told that DNP-graduates are able to “do” implementation science—when, in fact, DNP graduates are *not* prepared to do this—it undermines the importance and focus of the DNP degree. Skills in EBPQI are desperately needed in health care (ie, what DNP graduates *should* be able to do)^4^; however, this inadvertently gets overshadowed when research is consistently touted as the “end goal.”

Additionally, we disagree that “the evolution of the DNP role suggests value in the inclusion of research training for the conduct of translational research.” Again, translational research is just that, *research* studies at all levels T1-T4 (from bench to population).^5^

Why should the full partnership goal always be focused on the conduct of research? This point of view continues to place the DNP-prepared nurse or APRN in the “helper” role to the PhD-prepared nurse who is the expert on conducting and leading research efforts. This also perpetuates the role confusion for students, faculty, and our practice partners. Nursing already has a research-focused doctorate (PhD); if nurses are interested in conducting research, a PhD degree should be pursued. What nursing needs is a *practice doctorate* with nurses having the skills and ability to translate research findings into practices through EBP and QI methodologies—this is why the DNP degree was created (and is needed). We don't see the benefit in having two nursing degrees in which the end goal is research. There needs to be clear understanding of the differences between PhD and DNP preparation, as well as what implementation science is (and is not). Until we can achieve more clarity and parity, true equitable partnerships where each brings their expertise and leadership to the partnership will continue to be a challenge.

**—Julee Briscoe Waldrop, DNP, FNP-BC, PNP-BC, FAANP, FAAN and Staci S. Reynolds, PhD, RN, ACNS-BC, CCRN, CNRN, CPHQ, FAAN**
